# Lower fat-free mass is independently linked to restless legs syndrome in men: a cross-sectional PSG–BIA study

**DOI:** 10.3389/fneur.2026.1749591

**Published:** 2026-03-06

**Authors:** Heewon Bae, Hea Ree Park, Hosung Kim, Eun Yeon Joo

**Affiliations:** 1 Department of Neurology, Inje University College of Medicine, Ilsan Paik Hospital, Goyang, Republic of Korea; 2 Department of Neurology, Neuroscience Center, Samsung Medical Center, Sungkyunkwan University School of Medicine, Seoul, Republic of Korea; 3 Mark and Mary Stevens Neuroimaging and Informatics Institute, Keck School of Medicine, University of Southern California, Los Angeles, CA, United States

**Keywords:** bioelectrical impedance analysis, fat-free mass index, restless legs syndrome, sarcopenia, skeletal muscle index

## Abstract

**Objective:**

Restless legs syndrome (RLS) is a common sensorimotor disorder that disrupts sleep and quality of life. Sarcopenia—reduced skeletal muscle mass and function—has been linked to sleep disturbances, but its relationship with RLS remains unclear. We examined whether sarcopenia is associated with RLS, with a focus on sex-specific effects.

**Methods:**

We conducted a cross-sectional analysis of 5,752 adults who underwent both type-I polysomnography (PSG) and bioelectrical impedance analysis (BIA) at a tertiary sleep center. RLS was diagnosed by IRLSSG criteria. Sarcopenia was defined using skeletal muscle index (SMI) and fat-free mass index (FFMI) thresholds. Multivariable models adjusted for age, physical activity, caffeine/alcohol intake, and apnea–hypopnea index (AHI).

**Results:**

RLS prevalence was 6.6% in females and 2.9% in males. Sarcopenia was more frequent in the RLS group than in non-RLS (10.6 vs. 6.8%), particularly among males (8.7 vs. 3.2%). In males, lower SMI and FFMI were independently associated with higher odds of RLS; sex interaction for FFMI was significant.

**Conclusions:**

Reduced muscle mass is independently associated with RLS in men, suggesting a male-specific muscle phenotype relevant to RLS pathophysiology. Incorporating BIA-based screening and muscle-preserving interventions may benefit the management of male patients with RLS.

## 1 Introduction

Restless legs syndrome (RLS) is a sensorimotor neurological disorder prevalent in approximately 5%−20% of adults (1). It manifests as an irresistible urge to move the legs that worsens during periods of rest, particularly in the evening. Consequent sleep disturbances and disruptions in quality of life associated with these symptoms emphasize the critical need for effective RLS treatment (2, 3). Although pharmacological interventions are currently the established treatment for RLS, there is growing interest in various non-pharmacological approaches. Diverse therapeutic attempts being explored include repetitive transcranial magnetic stimulation, exercise, compression devices, counter strain manipulation, infrared therapy, and standard acupuncture (4). Among these, exercise has

the potential to be highly effective in alleviating RLS symptoms (5). Several studies have reported the efficacy of muscle-strengthening exercises in improving symptoms such as leg discomfort and sleep disturbances, while also improving the overall wellbeing of patients with RLS (6). This effectiveness is attributed to the role of physical training in improving oxygen delivery to muscles, suggesting an association between muscle mass and alleviation of RLS symptoms (7).

Sarcopenia, characterized by an age-related decline in skeletal muscle mass, has emerged as a major medical and economic concern in aging populations (8). Studies have indicated an association between sarcopenia and sleep disorders, with reductions in sleep duration and quality, and disturbances in sleep cycles observed in adults with sarcopenia (9). This can be attributed to the role of sleep in muscle protein metabolism (10). Considering the inhibition of catabolic hormone pathways and enhancement of anabolic pathways in skeletal muscles, we hypothesized a potential association between sarcopenia and RLS. In this study, we measured body composition, including muscle mass, using bioelectrical impedance analysis (BIA), and investigated the association between RLS and sarcopenia.

## 2 Materials and methods

### 2.1 Study population

**Figure 1:**
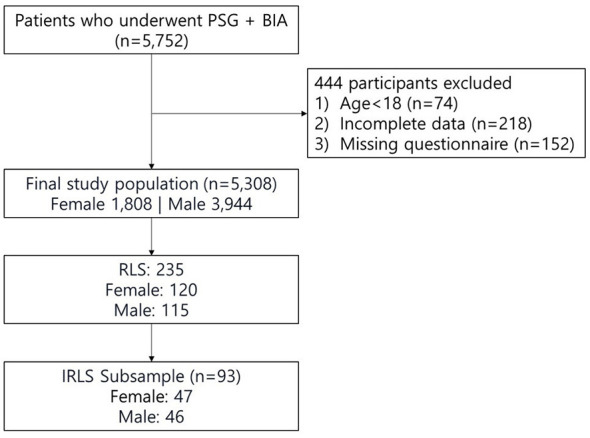
Flow diagram of participant selection. Flowchart illustrating the selection of participants included in the analysis.

This cross-sectional study utilized data from the Department of Neurology at Samsung Medical Center, Seoul, Korea. The study included patients who visited the sleep center for sleep disorders and completed overnight type I polysomnography (PSG) and BIA from December 2017 to December 2019. These patients completed sleep questionnaires because they came with sleep-related problems. Participants aged < 18 years, those unable to stand for an elementary body composition test, and those who did not undergo PSG and BIA were excluded. A total of 5,752 patients were included in the study (Figure 1). Approval for the study was obtained from the institutional review board of Samsung Medical Center, and informed consent was waived owing to the non-interventional observational nature of the study (approval number: 2023-05-012).

### 2.2 RLS diagnosis

The diagnosis of RLS was established by a neurologist specializing in sleep medicine using the updated diagnostic criteria of the International Restless Legs Syndrome Study Group (11). Concurrently, the presence of periodic limb movements in sleep (PLMS), which serves as a supplementary clinical feature for diagnosing RLS, was assessed using overnight PSG. Periodic limb movements in sleep (PLMS) were scored according to the official standards of the World Association of Sleep Medicine (12). Among the 235 RLS patients, 93 patients who consented to questionnaire completion underwent International Restless Legs Study Group Severity Rating Scale (IRLS) measurement to evaluate symptom severity.

Iron-related parameters, including serum iron, ferritin, and total iron-binding capacity, were available only in a subset of patients with RLS and were not systematically measured in the non-RLS group. Therefore, iron status was not included as a covariate in the primary regression models to avoid selection bias due to incomplete and non-uniform measurements. Similarly, detailed medication data were not consistently available across the cohort and were not included in multivariable analyses.

### 2.3 PSG

All participants underwent an overnight type I PSG, which involved six-channel electroencephalography, two-channel electrooculography, and a minimum of four-channel electromyography to monitor various muscle groups, including the chin, intercostals, and right and left anterior tibialis. Respiratory parameters were monitored using nasal pressure transducers, thermistors, thoracic and abdominal respiratory effort belts, and pulse oximetry to assess oxygen saturation levels. This allowed the analysis of sleep stages, arousals, apneas/hypopneas, and rapid-eye-movement sleep without atonia. The PSG assessments were performed manually according to the criteria established by the American Academy of Sleep Medicine scoring manual (13). Trained PSG technicians conducted PSG, and acquired data were reviewed by certified sleep specialists.

### 2.4 BIA parameters

The human body is composed of water, proteins, fat, and minerals, all of which exhibit different bioelectrical impedances. The BIA was performed using an InBody 770 apparatus (Biospace, Seoul, Korea) during the PSG examinations. This device uses a direct segmental multifrequency BIA technique to assess body composition, using 30 impedance readings taken at six frequencies (1, 5, 50, 250, 500, and 1,000 kHz) across the arms, legs, and trunk. The BIA is a convenient and non-invasive procedure. Following a 3-h fast, participants emptied their bladders, grasped the detection handles, and remained stationary, while data were automatically collected. The parameters measured encompassed body mass index (BMI), skeletal muscle index (SMI), visceral fat area (VFA), skeletal muscle mass (SMM), fat-free mass index (FFMI), and fat mass index (FMI). Sarcopenia was defined as an SMI of < 7 and < 5.7 kg/m^2^ for males and females, respectively, and an FFMI of < 18 and < 15 kg/m^2^ for males and females, respectively (14–16). Although current consensus definitions of sarcopenia include assessments of muscle strength and physical performance, the present study focused on muscle mass–based indices derived from BIA because functional measures were not available in this retrospective cohort.

### 2.5 Statistical analysis

Baseline characteristics were summarized as numbers and proportions for categorical variables and as means with standard deviations for continuous variables. Group comparisons between RLS and non-RLS participants were performed using the chi-square or Fisher's exact test for categorical variables and the independent *t*-test or Wilcoxon rank-sum test for continuous variables, as appropriate.

Multivariable logistic regression analyses were conducted to examine the independent association between body composition indices and the risk of RLS. Models were adjusted for potential confounders, including age (years), physical activity (times/week), caffeine and alcohol consumption, and apnea–hypopnea index (AHI). To evaluate sex-specific effects, analyses were stratified by sex, and an interaction term (FFMI × Sex) was further tested in a stepwise model to assess potential moderating effects. Model discrimination was evaluated using the area under the receiver operating characteristic curve (AUC).

Both continuous variables (e.g., SMI, FFMI) and categorical definitions of sarcopenia based on established cut-off values (SMI < 7.0 kg/m^2^ for males and < 5.7 kg/m^2^ for females; FFMI < 18 kg/m^2^ for males and < 15 kg/m^2^ for females) were entered into the regression models.

To explore relationships between body composition and symptom burden, Pearson's correlation analyses were performed between BIA parameters and the International Restless Legs Syndrome (IRLS) severity scores in patients with RLS, stratified by sex.

All tests were two-tailed, and a *p*-value < 0.05 was considered statistically significant. Statistical analyses were performed using R version 4.3.1 (R Foundation for Statistical Computing, Vienna, Austria).

## 3 Results

### 3.1 Prevalence of RLS and general characteristics

In this study, the prevalence of RLS was approximately 4.1%, with a higher prevalence in females (6.6%) than in males (2.9%). The mean age of the RLS group was 61.65 ± 9.72 years and 60.86 ± 11.85 years for females and males, respectively, which was higher than that of the control group for males (54.31 ± 14.44 years) and females (51.08 ± 14.45 years). In females with RLS, lower alcohol consumption was observed (*p* = 0.003), while in males with RLS, lower alcohol and caffeine consumption were observed (*p* = 0.002 and *p* < 0.001, respectively) along with higher levels of physical activity (*p* = 0.001). Additionally, BMI in males with RLS (24.45 ± 2.61) was lower than in the control group (26.51 ± 4.09, *p* < 0.001). The Pittsburgh Sleep Quality Index (PSQI) and insomnia sleep index (ISI) scores were higher in both males and females with RLS than in the control group, indicating greater sleep disturbances. Among males, the AHI was significantly lower in the RLS group (6.81 ± 12.87). The periodic leg movement index was significantly higher in male RLS patients (42.03 ± 43.63) compared to non-RLS males (24.12 ± 27.73; *p* < 0.001), while no significant difference was observed in females (Table 1). Iron-related parameters showed significant sex differences in RLS patients. Males had higher serum iron (106.69 ± 38.29 vs. 94.25 ± 33.39 μg/dl, *p* = 0.046) and ferritin levels (206.93 ± 119.09 vs. 129.46 ± 120.85 ng/ml, *p* = 0.001) compared to females. TIBC levels showed no significant difference between sexes (293.09 ± 51.32 μmol/L in males vs. 304.14 ± 41.32 μmol/L in females, *p* = 0.172; Supplementary Table S1).

**Table 1:** Characteristics of study subjects.

**Variable**	**Female**			**Male**		
	**Non-RLS (*****n*** = **1,688)**	**RLS (*****n*** = **120)**	***p-*** **value**	**Non-RLS (*****n*** = **3,839)**	**RLS (*****n*** = **115)**	***p-*** **value**
Age (years)	54.31 ± 14.44	61.65 ± 9.72	**< 0.001** ^*^	51.08 ± 14.45	60.86 ± 11.85	**< 0.001** ^*^
Hypertension	52 (3.1)	4 (3.3)	0.436	1,097 (28.6)	35 (30.4)	0.882
CVD	7 (0.4)	1 (0.8)	0.398	65 (1.7)	3 (2.6)	0.756
Psychiatric disorder	250 (14.8)	26 (21.7)	0.11	202 (5.3)	9 (7.8)	0.319
Alcohol	456 (27.0)	22 (18.3)	**0.003** ^*^	2,411 (62.8)	55 (47.8)	**0.002** ^*^
Caffeine	1,035 (61.3)	68 (56.7)	0.258	2,967 (77.3)	73 (63.5)	**< 0.001** ^*^
Physical activity (num/week)	3.59 ± 1.56	3.92 ± 1.41	0.055	3.48 ± 1.63	4.06 ± 1.75	**0.001** ^*^
**Anthropometric measurements**						
Height, cm	157.82 ± 6.12	156.6 ± 5.25	**0.033** ^*^	171.29 ±7.49	169.42 ± 5.71	**0.008** ^*^
Weight, kg	60.5 ± 11.9	59.61 ± 9.22	0.421	78.04 ± 13.83	70.24 ± 8.81	**< 0.001** ^*^
Body mass index (kg/m^2^)	24.31± 4.64	24.30 ± 3.5	0.996	26.51 ± 4.09	24.45 ± 2.61	**< 0.001** ^*^
NC, cm	33.73 ± 3.89	34.08 ± 2.62	0.333	39.52 ± 13.68	38.0 ± 3.84	0.235
WC, cm	60.5 ± 11.9	59.61 ± 9.22	0.421	92.74 ± 13.07	89.78 ± 8.25	**0.016** ^*^
HC, cm	92.86 ± 11.39	93.03 ± 11.02	0.874	97.05 ± 13.26	94.15 ± 5.57	**0.02** ^*^
**Questionnaires**						
PSQI-K	9.56 ± 4.4	11.98 ± 4.22	**< 0.001** ^*^	7.52 ± 4.07	9.89 ± 4.82	**< 0.001** ^*^
ISI	9.56 ± 4.4	11.98 ± 4.22	**0.004** ^*^	10.71 ± 6.29	13.34 ± 6.29	**< 0.001** ^*^
ESS	8.64 ± 5.2	8.04 ± 5.23	0.226	9.53 ± 5.04	10.06 ± 5.09	0.265
K-BDI	16.13 ± 9.65	17.51 ± 10.09	0.162	12.22 ± 8.97	12.90 ± 8.63	0.439
**PSG**						
Total sleep time (min)	359.7 ± 66.98	350.79 ± 60.94	0.157	346.06 ± 66.59	335.70 ± 66.12	0.1
Sleep latency (min)	17.79 ± 25.21	18.25 ± 26.15	0.844	10.85 ± 16.81	9.8 ± 13.35	0.506
REM sleep latency (min)	120.7 ± 71.65	132.65 ± 72.73	0.079	107.06 ± 65.04	105.91 ± 65.78	0.855
Wakefulness after sleep onset (min)	67.57 ± 50.22	74.64 ± 59.13	0.141	57.21 ± 49.11	77.79 ± 60.48	**0.024** ^*^
N1 (%)	16.58 (9.85)	15.11 (6.9)	0.108	24.12 (14.47)	21.6 (11.07)	0.064
N2 (%)	56.24 (10.98)	59.96 (12.12)	**< 0.001** ^*^	52.98 (12.27)	58.47 (11.13)	**< 0.001** ^*^
N3 (%)	7.77 (8.83)	6.3 (8.17)	0.078	4.42 (6.49)	2.31 (4.92)	**0.001** ^*^
REM (%)	19.41 (7.08)	18.62 (6.98)	0.238	18.49 (6.93)	17.62 (7.48)	0.186
Sleep efficiency (%)	80.92 ± 12.86	79.51 ± 13.33	0.25	81.61 ± 12.34	79.55 ± 13.52	0.079
Arousal index (/h)	20.90 ± 11.35	21.19 ± 9.05	0.787	29.72 ± 16.84	26.52 ± 13.2	**0.044** ^*^
Apnea-hypopnea index (/h)	4.1 ± 10.00	2.36 ± 4.86	0.059	13.52 ± 20.22	6.81 ± 12.87	**< 0.001** ^*^
Periodic leg movement index (/h)	21.27 ± 23.32	25.00 ± 23.46	0.184	24.12± 27.73	42.03 ± 43.63	< 0.001

RLS, restless legs syndrome; CVD, cardiovascular disease; NC, neck circumference; HC, hip circumference; WC, waist circumference; PSQI, Pittsburgh Sleep Quality Index-Korean Version; ISI, insomnia sleep index; ESS, Epworth Sleepiness Scale; K-BDI, Korean Version-Beck Depression Inventory; REM, rapid eye movement. Bold values indicate statistical significance at *p* < 0.05.

### 3.2 Association between BIA parameters and RLS

**Figure 2:**
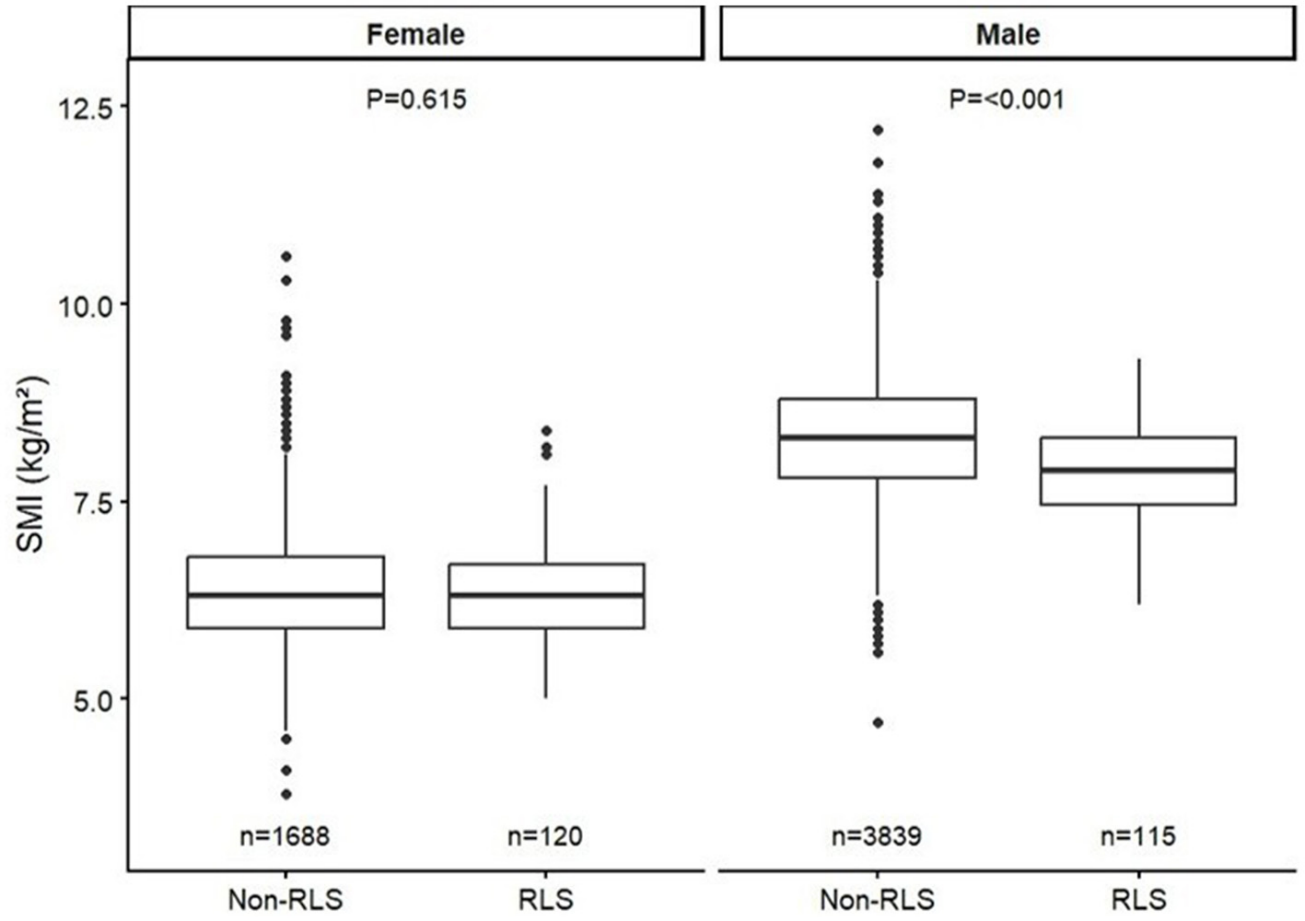
Sex-specific differences in SMI and FFMI according to RLS status. Box plots comparing skeletal muscle index (SMI, kg/m^2^; left) and fat-free mass index (FFMI, kg/m^2^; right) between participants with and without restless legs syndrome (RLS), stratified by sex. Sample sizes for each subgroup are indicated within the figure. Data are presented as medians with interquartile ranges; circles denote outliers.

When comparing the BIA parameters related to sarcopenia, specifically the SMI and FFMI, no significant differences were observed between the two groups in females. However, in males, both SMI and FFMI indicated significant differences between the RLS and control groups, with values of 7.89 ± 0.63 and 8.31 ± 0.78 for SMI and 18.52 ± 1.41 and 19.48 ± 1.74 for FFMI, respectively (Figure 2).

In multivariable logistic regression adjusting for age, BMI, AHI, exercise, caffeine, and alcohol use, lower BMI was associated with a reduced risk of RLS in males (OR = 0.998, 95% CI 0.995–0.999, *p* = 0.043). In multivariable models, higher SMI and FFMI were independently associated with lower odds of RLS in men, whereas no associations were observed in women. When defining sarcopenia in males using criteria of SMI < 7 (kg/m^2^) and an FFMI < 18 (kg/m^2^), sarcopenia increased the risk of RLS with an OR of 1.065 (95% CI: 1.020–1.112) for SMI and 1.025 (95% CI: 1.004–1.047) for FFMI (Table 2).

**Table 2:** Multivariable logistic regression analysis of RLS according to BIA parameters.

**Parameter**	**OR**	***t***	***p-*value**	**95% CI**
**BMI (25 kg/m**^2^≥**)**				
Female	0.993	−0.356	0.722^*^	0.958–1.029
Male	0.970	−3.769	0.0002^*^	0.954–0.985
**BMI (cont.)**				
Female	0.999	−0.321	0.749	0.995–1.004
Male	0.998	−2.022	**0.043** ^*^	0.95–0.999
**SMI (Female**,<**5.7 kg/m**^2^**; Male**,<**7.0 kg/m**^2^**)**				
Female	0.994	−0.247	0.805	0.949–1.041
Male	1.065	2.857	**0.004** ^*^	1.004–1.077
**SMI (Cont)**				
Female	0.999	−0.030	0.976	0.976–1.024
Male	0.987	−2.311	**0.021** ^*^	0.976–0.998
**VFA**				
Female	0.999	−0.217	0.829	0.99–1.000
Male	0.999	−0.932	0.351	0.999–1.000
**SMM/VFA**				
Female	0.942	−0.726	0.468	0.999–1.000
Male	1.020	0.933	0.351	0.99–1.000
**FFMI (Female**,<**15 kg/m**^2^**; Male**,<**18 kg/m**^2^**)**				
Female	1.005	0.244	0.807	0.967–1.044
Male	1.025	2.367	0.018^*^	1.004–1.047
**FFMI (Cont.)**				
Female	0.996	−0.747	0.455	0.984–1.007
Male	0.994	−2.504	**0.012** ^*^	0.988–0.999
**FMI**				
Female	0.999	−0.199	0.842	0.994–1.005
Male	0.995	−1.346	0.178	0.994–1.001

Adjustment for age (years), physical activity (n/week), caffeine, alcohol consumption, and apnea-hypopnea index.

BIA, bioelectrical impedance analysis; BMI, body mass index; SMI, skeletal muscle index; FFMI, fat-free mass index; FMI, fat mass index; VFA, visceral fat area; SMM, skeletal muscle mass; OR, odds ratio; CI, confidence interval. Bold values indicate statistical significance at *p* < 0.05.

**Figure 3:**
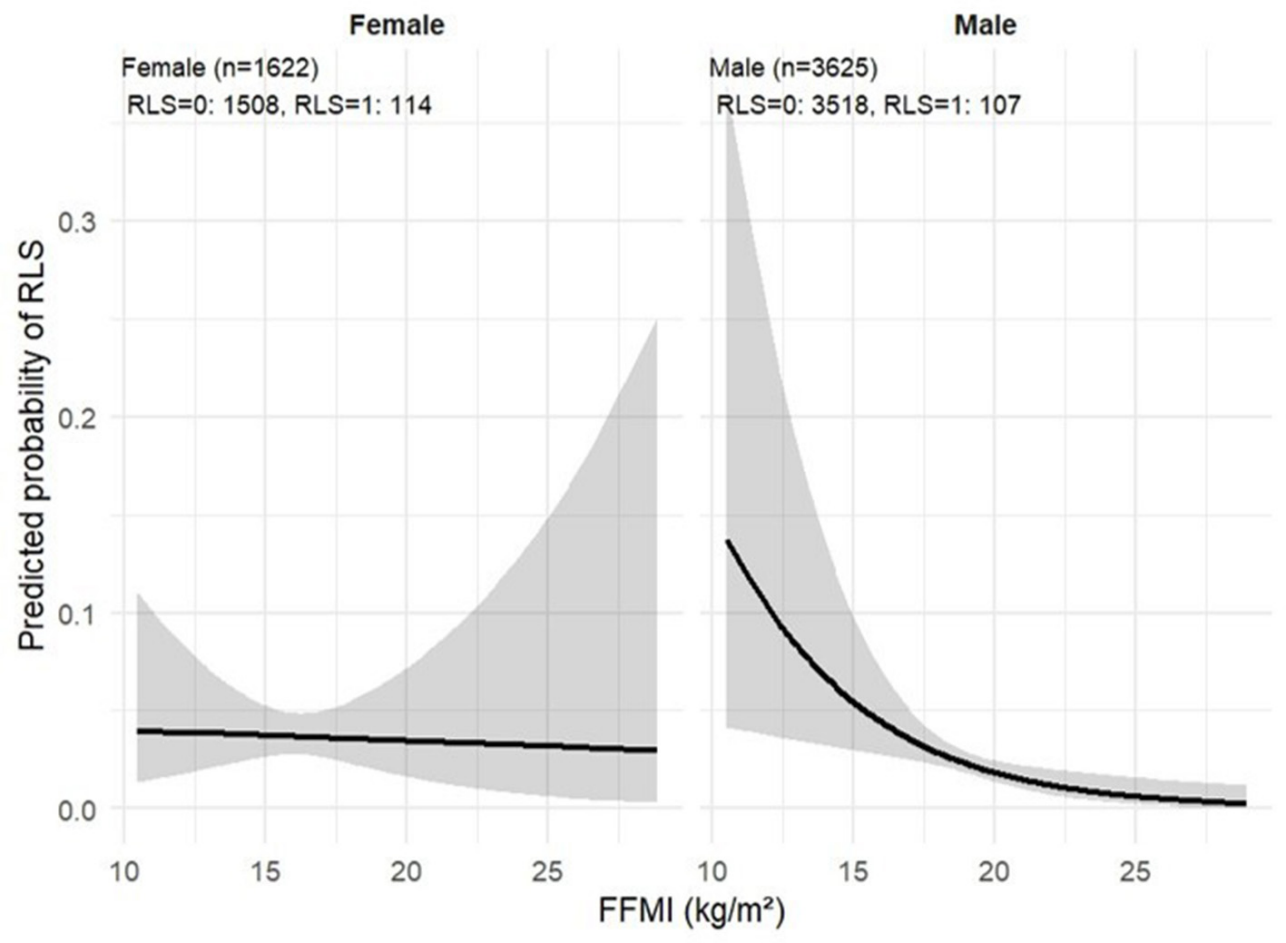
Interaction between FFMI and sex on RLS risk. Interaction plot illustrating the sex-specific association between fat-free mass index (FFMI, kg/m^2^) and the probability of restless legs syndrome (RLS) based on a multivariable logistic regression model including an FFMI × sex interaction term. Lines represent predicted probabilities with 95% confidence intervals. Sample sizes (*n*) for males and females are shown in each panel.

A stepwise model including the FFMI × sex interaction confirmed a significant sex-specific effect (interaction *p* = 0.037): the FFMI effect was protective in men (OR 0.798, 95% CI 0.685–0.931, *p* = 0.004) but not in women (OR 0.984, 95% CI 0.817–1.184, *p* = 0.863; Table 3, Figure 3).

**Table 3:** Stepwise logistic regression for the association between FFMI and RLS.

**Term**	**Model 1 (Base)**	**Model 2 (+FFMI)**	**Model 3 (FFMI × Sex)**
FFMI (per 1 kg/m^2^)	–	0.859 (0.752–0.986), *p* = 0.029	0.984 (0.817–1.184), *p* = 0.863
FFMI × male	–	–	0.811 (0.668–0.988), *p* = 0.037
FFMI effect in Women	–	0.859 (0.752–0.986), *p* = 0.029	0.984 (0.817–1.184), *p* = 0.863
FFMI effect in Men	–	0.859 (0.752–0.986), *p* = 0.029	0.798 (0.685–0.931), *p* = 0.004
AUC	0.76	0.762	0.764

Model 1 = Base model adjusted for age, BMI, AHI, exercise, caffeine, and alcohol use.

Model 2 = Model 1 + FFMI.

Model 3 = Model 2 + interaction term (FFMI × sex).

In Model 3, the FFMI coefficient represents the effect in women; the effect in men = FFMI + FFMI × male (linear combination).

Analysis of the relationship between body composition parameters and RLS severity revealed sex-specific correlations. In males, VFA showed a significant negative correlation with RLS severity (*r* = −0.388, *p* < 0.05), while SMM/VFA ratio showed a positive correlation (*r* = 0.31, *p* < 0.05). In females, the lack of statistically significant associations between most BIA parameters and RLS should be interpreted with caution, as the female RLS subgroup was relatively small, limiting statistical power. Notably, FFMI showed a significant association with IRLS severity in females, suggesting a potential sex-specific relationship that warrants replication and validation in larger female cohorts (Table 4).

**Table 4:** Linear regression of IRLS severity on FFMI.

**Term**	**β (95% CI)**	***p*-value**
Overall (*n* = 93)	−0.99 (−2.43, 0.44)	0.18
Male (*n* = 46)	−0.74 (−2.02, 0.54)	0.25
Female (*n* = 47)	2.86 (0.16, 5.55)	**0.038**

Bold values indicate statistical significance at *p* < 0.05.

The absence of significant associations in females may be partly attributable to limited power due to cohort imbalance; however, biological and clinical sex-specific factors should also be considered. Sex hormone–related differences in muscle metabolism and anabolic signaling, as well as sex-related variation in iron regulation and symptom perception, may influence RLS expression and severity. Additionally, women may present with less motor-dominant or atypical RLS symptoms, potentially contributing to diagnostic heterogeneity. These factors may underlie the observed sex-specific patterns and highlight the need for further investigation in larger, sex-balanced cohorts.

BMI, SMI, FFMI, and FMI showed no significant correlations with RLS severity in either sex (Supplementary Table S2).

## 4 Discussion

To our knowledge, this is the first study to investigate the association between body composition and RLS using BIA. By integrating objective body composition measures, this study provides novel evidence that reduced skeletal muscle and fat-free mass are independently associated with RLS, particularly in men. These findings suggest that alterations in skeletal muscle and fat-free compartments may be associated with increased RLS susceptibility, particularly in men, suggesting a possible link between peripheral muscle status and central mechanisms involved in sensorimotor regulation. Moreover, the observed sex-specific association raises the possibility that hormonal, metabolic, or dopaminergic differences between men and women could influence this muscle–neural relationship.

Previous research has mainly focused on the association between body mass index (BMI) and RLS rather than directly assessing muscle mass. Although some studies have reported a positive relationship between higher BMI and RLS, others have found no association. A meta-analysis of 15 studies involving 197,204 participants found that overweight and obesity increased the OR for RLS to 1.44 (95% CI: 1.31–1.58) and 1.29 (95% CI: 1.22–1.36), respectively (17). Conversely, a large Turkish population-based study of 3,234 adults reported no difference in RLS prevalence according to BMI (18). In our study, BMI showed a sex-specific pattern—in males, a higher BMI was protective against RLS (Table 2). This discrepancy may partly reflect racial or genetic factors but also underscores the limitation of BMI as a crude index that does not distinguish between fat and lean components. The apparent “BMI paradox” may indicate that, in some populations, higher BMI corresponds to greater muscle mass rather than obesity, highlighting the need for direct body composition measurements such as skeletal muscle index (SMI) or fat-free mass index (FFMI) (15).

In this context, we evaluated body composition using BIA to clarify its association with RLS. Indices representing lean mass, such as SMI and FFMI, were significantly lower in males with RLS than in controls, while no significant difference was observed in females (Figure 2). In contrast, fat-related parameters, including visceral fat area (VFA) and fat mass index (FMI), did not differ significantly between groups. Moreover, among males, VFA was inversely correlated with RLS severity (*r* = −0.388, *p* < 0.05), whereas the skeletal muscle–to–visceral fat ratio (SMM/VFA) showed a positive correlation (*r* = 0.31, *p* < 0.05; Supplementary Table S2).

These observations imply that the relative balance between muscle and fat tissue, rather than total adiposity, may play a role in the clinical expression of RLS in men. Although the underlying mechanisms remain speculative, visceral fat is known to promote systemic inflammation and oxidative stress, while skeletal muscle has anti-inflammatory and neurotrophic properties mediated by myokines such as irisin and brain-derived neurotrophic factor (BDNF) (19, 20). Chronic inflammation and oxidative stress can disrupt dopaminergic neurotransmission in the basal ganglia, a core mechanism implicated in RLS pathophysiology. Furthermore, reduced lean mass may reflect impaired mitochondrial function and oxygen utilization in skeletal muscle, potentially contributing to the peripheral hypoxia previously described in RLS (21). These mechanistic links remain hypothetical and warrant validation through physiological and longitudinal studies.

The interaction between muscle physiology and sleep regulation may further explain this association. Several population-based studies have reported that short sleep duration and poor sleep efficiency are associated with an increased risk of sarcopenia, particularly among men (17, 22). A systematic review found that short sleep (< 6 h) or low sleep efficiency tended to be more strongly associated with sarcopenia in males (OR 1.61; 95% CI: 0.82–3.16; *Q* = 11.80; *p* = 0.0189) than in females (OR 0.77; 95% CI: 0.29–2.03; *Q* = 21.35; *p* = 0.0003) (21). Sleep deprivation may reduce testosterone and impair the activation of insulin-like growth factor-1 (IGF-1), a key mediator of muscle protein synthesis, leading to diminished myofibrillar protein turnover (9, 17, 23). In addition, elevated cortisol levels caused by poor sleep can suppress the IGF-1 signaling pathway and promote systemic inflammation through cytokine release (23). Circadian disruption and reduced melatonin secretion may also impair mitochondrial homeostasis and muscle regeneration, further exacerbating sarcopenic processes. Conversely, decreased muscle mass and strength have been linked to reduced slow-wave sleep and poorer sleep quality, suggesting a bidirectional interaction between muscle health and sleep disturbance rather than a unidirectional effect.

Our results revealed that the association between sarcopenia and RLS was significant only in men. This sex-specific pattern may be explained by both biological and methodological factors. Males exhibited higher serum iron and ferritin levels than females, consistent with prior evidence that iron deficiency contributes more prominently to RLS in women (9, 24). Accordingly, muscle- and metabolism-related pathways may be relatively more relevant in men, whereas iron dysregulation and hormonal changes could predominate in women. Experimental work has shown that men experience a measurable reduction in testosterone—a key anabolic hormone—in response to sleep fragmentation, even after short periods of insufficient sleep (25). Because RLS is characterized by PLMS-related arousals, men may be more vulnerable to reductions in nocturnal anabolic signaling when sleep continuity is disrupted (26). Furthermore, men tend to exhibit a greater decline in lean mass when physical activity decreases, suggesting that RLS-related discomfort or fatigue may amplify muscle-metabolic consequences in males (27). Future studies with more balanced cohorts are warranted to confirm these sex-specific associations.

From a clinical perspective, our findings suggest that assessing muscle health may have practical implications for RLS management, particularly in male patients. Structured resistance and aerobic exercise programs that enhance muscle strength, peripheral oxygenation, and dopaminergic stability could be explored as potential adjunctive strategies, although these remain to be verified in interventional trials (4–8). These results highlight the need to view RLS not solely as a central dopaminergic disorder but as a condition potentially influenced by systemic metabolic and muscular factors (28, 29).

This study had several limitations. First, the cross-sectional design restricted our ability to identify causal relationships between sarcopenia and RLS. Reduced muscle mass in RLS may reflect both secondary effects of PLMS-related sleep fragmentation and physical inactivity, as well as muscle-related mechanisms relevant to RLS pathophysiology. Although physical activity was adjusted for in multivariable models, lower SMI and FFMI remained independently associated with RLS in men. These findings are consistent with a bidirectional relationship between muscle loss and RLS, rather than a purely secondary phenomenon, while causal inference cannot be made due to the cross-sectional design. Second, all participants were recruited from a tertiary sleep center, and therefore both the RLS and non-RLS groups represent a sleep-disorder clinic–oriented population rather than healthy community controls. In particular, the non-RLS group likely included individuals with other sleep disorders, especially obstructive sleep apnea. Although apnea–hypopnea index was adjusted for in all multivariable models, residual confounding related to referral bias cannot be fully excluded, and the generalizability of these findings to the general population may be limited. Specifically, the study was particularly noteworthy because the non-RLS group included patients with Obstructive sleep apnea (OSA) to account for potential selection bias because of OSA, a multivariate logistic regression analysis was performed, adjusting for AHI. However, future comparisons should include data from healthy participants.

Finally, the relatively low proportion of female participants in our study might explain why an association between sarcopenia and RLS was predominantly observed in males. Further research with a larger female cohort is required to explore these potential sex-related differences.

In addition, sarcopenia in the present study was defined solely based on muscle mass indices derived from BIA. According to current consensus definitions such as EWGSOP2, sarcopenia should ideally be diagnosed by combining measures of muscle mass with functional assessments, including muscle strength or physical performance. Accordingly, the present findings should be interpreted as associations with low muscle mass rather than clinically defined sarcopenia. Future studies incorporating both muscle mass and functional measures are needed to validate and extend these observations. Iron deficiency and medication use are well-established modifiers of RLS risk. However, in the present study, iron-related parameters and medication data were available only in a subset of patients, precluding their inclusion as covariates in the primary regression models. Iron deficiency and medication use are well-established modifiers of RLS risk. However, in the present study, iron-related parameters and medication data were available only in a subset of patients, precluding their inclusion as covariates in the primary regression models. Notably, the association between reduced muscle mass and RLS was predominantly observed in men, who exhibited relatively higher iron and ferritin levels than women in the available subgroup, suggesting that differences in iron status alone may not fully account for the observed sex-specific findings. Future studies incorporating systematic biochemical assessments and detailed medication histories are warranted.

Despite these limitations, this study has several strengths. It included a relatively large cohort that underwent both BIA and polysomnography, enabling objective assessment of body composition and sleep parameters. In addition, confounders such as OSA were carefully adjusted for in multivariable models. Together, these features enhance the validity of our findings and suggest that altered muscle composition and metabolism may represent modifiable risk markers for RLS, deserving further investigation in longitudinal and interventional research.

## 5 Conclusion

Our study revealed a significant association between sarcopenia and RLS in males, providing valuable insights into the pathophysiology of RLS. This underscores the need to evaluate muscle mass and exercise interventions for the comprehensive management of RLS. Considering the limitations of BMI, which does not capture body composition, BIA could be a helpful assessment tool for patients with RLS, along with proper exercise strategies to prevent or improve sarcopenia.

## Data Availability

The raw data supporting the conclusions of this article will be made available by the authors, without undue reservation.
