# Supplementary material for: Lower fat-free mass is independently linked to restless legs syndrome in men: a cross-sectional PSG–BIA study
Source: Front Neurol. 2026 Mar 6;17:1749591. doi: 10.3389/fneur.2026.1749591 (PMC13002388; doi:10.3389/fneur.2026.1749591)
Supplement: Supplementary file 1 [file Table_1.docx]

Supplementary Material

**Supplementary Table 1. Iron indices (sex difference)**

|  | Total (n=137) | Males (n=73) | Females (n=64) | P-value |
| --- | --- | --- | --- | --- |
| iron | 100.15±36.20 | 106.69±38.29 | 94.25±33.39 | 0.046 |
| ferritin | 161.44±125.56 | 206.93±119.09 | 129.46±120.85 | 0.001 |
| TIBC | 298.83±46.55 | 293.09±51.32 | 304.14±41.32 | 0.172 |

**Supplementaty Table 2. Correlation (IRLS vs BIA indices)**

|  | Total (n=93) | Males (n=46) | Females (n=48) |
| --- | --- | --- | --- |
| **BMI** | -0.05 | -0.17 | 0.09 |
| **BIA** |  |  |  |
| SMI | -0.168 | 0.009 | 0.083 |
| VFA, cm2 | -0.041 | -0.388* | 0.081 |
| SMM/VFA | -0.096 | 0.31* | -0.003 |
| FFMI | -0.109 | 0.14 | 0.081 |
| FMI | 0.031 | -0.85 | 0.067 |
